# Concurrent occurrence of electrochemical dissolution/deposition of cobalt–calcium phosphate composite†

**DOI:** 10.1039/d1ra05108c

**Published:** 2021-08-23

**Authors:** Eunji Pyo, Keunyoung Lee, Gi-Tae Park, Se-Young Ha, Seonhong Lee, Chung Soo Kim, Ki-Young Kwon

**Affiliations:** Department of Chemistry, Gyeongsang National University and Research Institute for Green Energy Convergence Technology Jinju 52828 South Korea kykwon@gnu.ac.kr; Analysis & Certification Center, Korea Institute of Ceramic Engineering & Technology Jinju 52851 South Korea

## Abstract

Amorphous cobalt–calcium phosphate composite (CCPC) films are electrochemically prepared on various electrodes by utilizing the solid phase of hydroxyapatite as a phosphate source. The CCPC film formation is surface process in which the dissolution of hydroxyapatite and the deposition of CCPC film concurrently occur on the electrode surface without the mass transfer of phosphate ions into the bulk solution. Elemental, crystallographic, and morphological analyses (EDX, ICP-AES, XPS, and XRD) indicate that the CCPC is composed of amorphous cobalt oxide with calcium and phosphate. The film exhibits durable oxygen evolution reaction (OER) catalytic properties under neutral and basic aqueous condition. Compared to using solution phase of phosphate source, our preparation method utilizing solid hydroxyapatite has advantage of preventing unnecessary chemical reaction between phosphate and other chemical species in bulk solution.

## Abstract 2

Solid phase of phosphate source (hydroxyapatite) is utilized for the electrochemical preparation of cobalt–calcium phosphate composite (CCPC) film.

## Graphical abstract

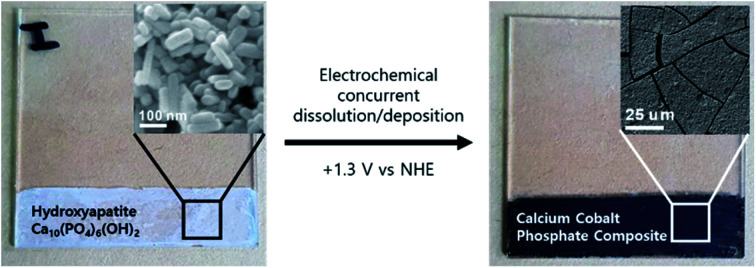

## Introduction

Metal phosphates are generally considered as chemicals possessing strong corrosion resistance.^1–3^ Therefore, various transition metal phosphates have been recently utilized for fields demanding high material stability and durability. For examples, so-called Co–Pi^4^ (amorphous cobalt oxide containing phosphate and potassium) and its derivatives are applied to oxygen evolution reaction (OER) catalysts, photoanodes, and cathodes for rechargeable batteries.^5–7^ It is known that most metal phosphates are insoluble salts except for alkaline or ammonium phosphate (NaH_2_PO_4_, K_2_HPO_4_, and NH_4_H_2_PO_4_*etc.*). Therefore, electrochemical film preparations of transition metal phosphates have been a considerable challenge because precipitation in bulk solution is faster than growth of thin film on electrodes. So far, aqueous solution of phosphoric acid or its alkaline/ammonium phosphate salts are used for the source of phosphate.^8–10^ To prevent precipitation of metal phosphates in bulk solution, electrocoating have been done under highly acidic electrolyte solution^11,12^ or low metal concentrations.^13^

In this study, we report on the novel preparation method of cobalt–calcium phosphate composite (CCPC) film in which solid hydroxyapatite^14^ (HAP, Ca_10_(PO_4_)_6_(OH)_2_) is utilized as a phosphate source. The CCPC film is formed by anodic oxidation of HAP loaded on ITO glass under aqueous Co^2+^ solution. We find that the deposition of the CCPC film concurrently proceeds with the dissolution of HAP. The usage of HAP enables the formation of the CCPC film without any unwanted precipitation in bulk solution. In addition, anodic deposition can be done under high concentration of cobalt ions (∼20 mM).

## Result and discussion

**Fig. 1:**
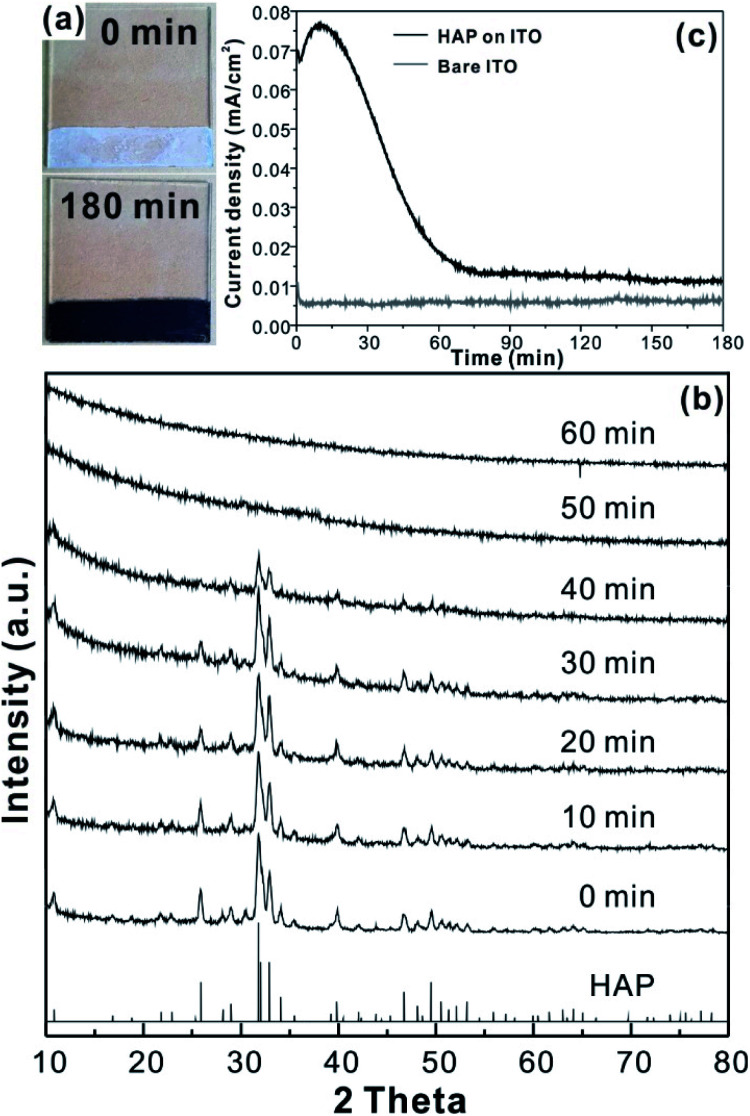
(a) Comparison of color between HAP (white) and the CCPC film (dark brown), (b) the change of XRD patterns during CCPC film formation, (c) current density profile for anodic oxidation at +1.3 V *vs.* NHE in 20 mM Co^2+^.

**Fig. 2:**
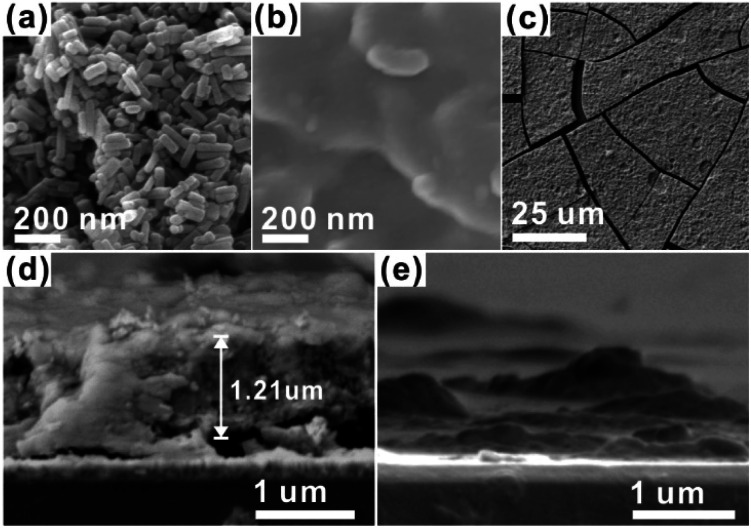
SEM images of (a) HAP, (b and c) CCPC film, vertical SEM images of (d) the CCPC film and (e) the film growth on bare ITO glass.

**Fig. 3:**
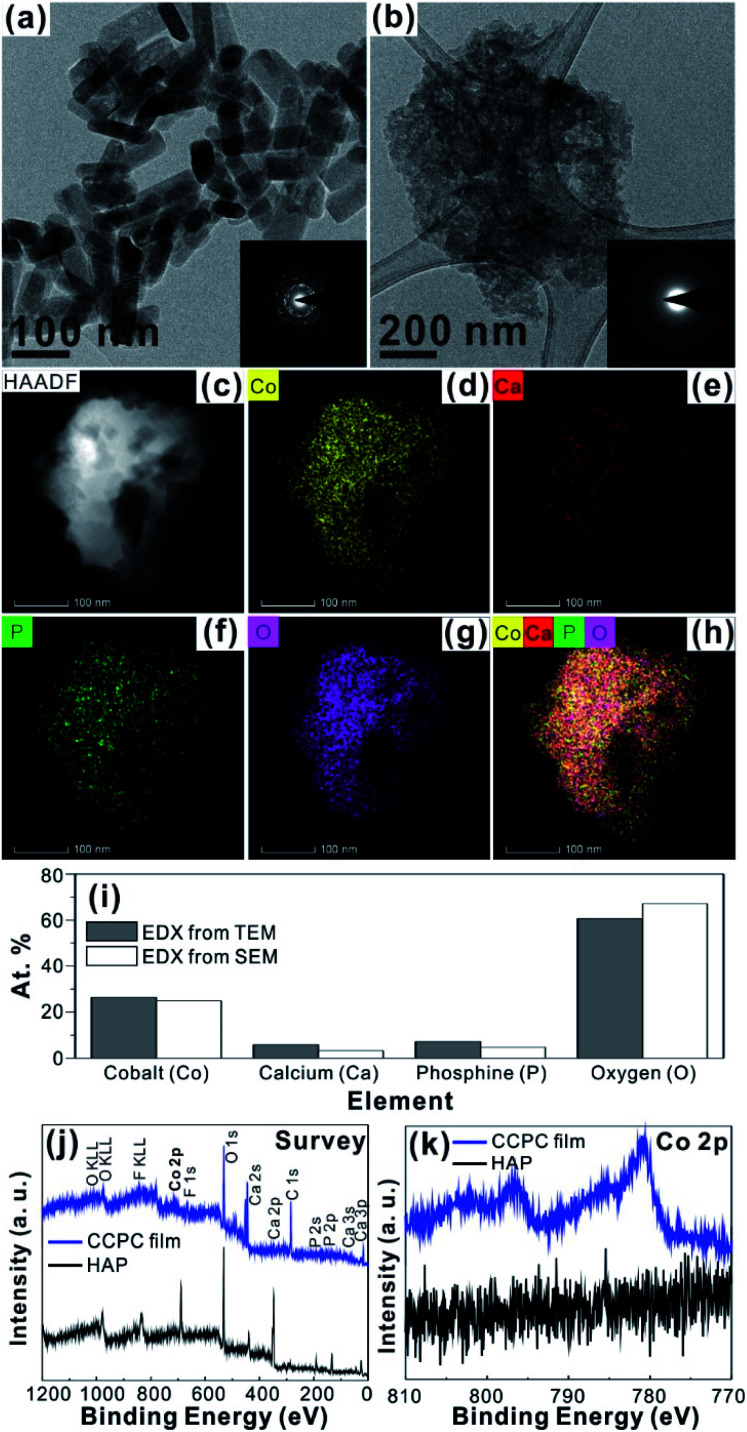
TEM images of (a) HAP and (b) CCPC film (inset; SAED of HAP and CCPC film), EDX elements mapping of CCPC film (c) TEM image, (d) Co, (e) Ca, (f) P, (g) O and (h) overlapping of Co, Ca, P and O, (i) atomic percent of CCPC film from TEM/SEM-EDX, survey (j) and Co2p (k) XPS spectra of pure HAP and CCPC film.

For the CCPC film preparation, the prepared HAP ink is loaded on ITO glass (details are included in ESI†). HAP is mainly composed of calcium cation/phosphate anion (Ca_10_(PO_4_)_6_OH_2_) and is a hardly soluble in neutral pH (*K*_sp_ = 2.35 × 10^−59^, 25 °C).^15^ The CCPC film is deposited on HAP loaded on ITO glass by anodic electrolysis at +1.3 V *vs.* NHE under 20 mM of aqueous Co^2+^ solution. White color of HAP on the ITO glass is changed into dark brown after 3 h electrolysis (Fig. 1a). In addition, Movie S1† includes the entire process of *in situ* formation of the CCPC film. White color of HAP is gradually changed into dark brown until 50 min. After 50 min, color change is hardly observed. Fig. 1b shows the sequential XRD patterns every 10 min time lapse for 1 hour. The crystallinity of HAP decreases as anodic electrolysis progresses. Finally, HAP crystallites no longer exist after 50 min. Fig. 1c shows the current profile during the formation of the CCPC film. The current density amounts to maximum value (0.077 mA cm^−2^) at 15 min, and gradually decreases to 0.016 mA cm^−2^. And current density reaches baseline value after ∼60 min with small fluctuation (±0.002 mA cm^−2^). The total charge flow for 60 min is 289 mC cm^−2^. In a control experiment, the current density under identical solution using bare ITO glass is a baseline level of less than ∼0.006 mA cm^−2^ (Fig. 1c). The color of bare ITO glass is changed into brownish yellow which can be obviously distinguished by the dark brown color of the CCPC film (Fig. S1†). Based on our observations; dramatic change of (i) color, (ii) XRD patterns, and (iii) current profile within 60 min during electrolysis, we conclude that the formation of the dark brown film and the dissolution of HAP concurrently occurred for the initial 60 min. It should be noticed that no precipitation resulting from reaction of phosphate ions of HAP with cobalt ions in bulk solution is observed during entire film formation. The morphological and elemental analyses of the CCPC film are studied by SEM/TEM and EDX/XPS/ICP-AES. Fig. 2a shows the SEM image of HAP loaded on an ITO glass before anodic oxidation. The HAP exhibits typical elongated hexagonal rod shape^16^ with approximately the length of 50–200 nm (Fig. 2a and 3a). After 3 h anodic oxidation, the elongated individual crystallites cannot be identified on the ITO glass (Fig. 2b). Instead, the electrodeposited film seems to be formed by coalesce of individual HAP crystallites. There are cracks on the film and the ITO glass can be identified through the cracks (Fig. 2c). The vertical SEM image of the CCPC film verifies that the overall film thickness is uniform with approximately 1.21 μm (Fig. 2d). In the case of the control film prepared using bare ITO glass, however, the film thickness is hardly measurable due to insufficient film formation (Fig. 2e). The TEM image of electrodeposited film (Fig. 3b) additionally confirms lack of particular shape. Moreover, no electron diffraction patterns is observed in the CCPC film (inset in Fig. 3a and b). The change of the crystallinity into amorphous phase is well accordance to the XRD data (Fig. 1b). STEM-EDX element mapping measurements (Fig. 3c–h) show that the cobalt, calcium, phosphorous, and oxygen species are well dispersed in the entire film. And the atomic compositions of elements are Co (26.35 at%), Ca (5.81 at%), P (7.18 at%) and O (60.65 at%). Additional elemental analysis for larger areas of the CCPC film is done using EDX housed in SEM (Fig. S2†). The composition of the film is Co (24.93 at%), Ca (3.22 at%), P (4.74 at%) and O (67.12 at%) (Fig. 3i). The atomic ratio of Co : Ca : P measured by ICP-AES is 1 : 0.29 : 0.21. All elemental analyses indicate that the major cation is Co instead of Ca. And the oxygen content much exceeds four times of the phosphorous content in the phosphate group (PO_4_), which indicates that the CCPC film is mainly composed of the mixture of amorphous cobalt oxide/phosphates with small amount of calcium ions. The surface composition of the CCPC film on the ITO is analysed by XPS. The CCPC survey spectrum verified the existence of calcium, cobalt, oxygen and phosphine (Fig. 2j and S3†). The binding energy at 780.7 and 790.5 eV (Fig. 2k) are attributed to Co2p_3/2_ and Co 2p_1/2_, respectively. The observation of both main Co2p_3/2_ and its weak shoulder peak imply that higher oxidation state of cobalt (Co^3+^ or Co^4+^) can be present in CCPC film.^17,18^

To investigate the details of the CCPC film formation process, we monitor the morphology change of HAP on ITO without applying potential under identical solution (50 mL of 20 mM Co^2+^ solution). No notable morphological change is observed by simple immersion of the sample into the cobalt solution for 3 h (Fig. S4b†). Additionally, we run electrolysis experiment under the electrolyte without Co^2+^ in which 20 mM of aqueous NaNO_3_ solution is chosen instead of 20 mM of Co(NO_3_)_2_ solution. The current density is negligible at +1.3 V *vs.* NHE (Fig. S5†), and morphology of HAP is not changed (Fig. S6b†). Therefore, we conclude that both bias (+1.3 V *vs.* NHE) and presence of Co^2+^ are essential for the formation of the CCPC film.

**Scheme 1:**
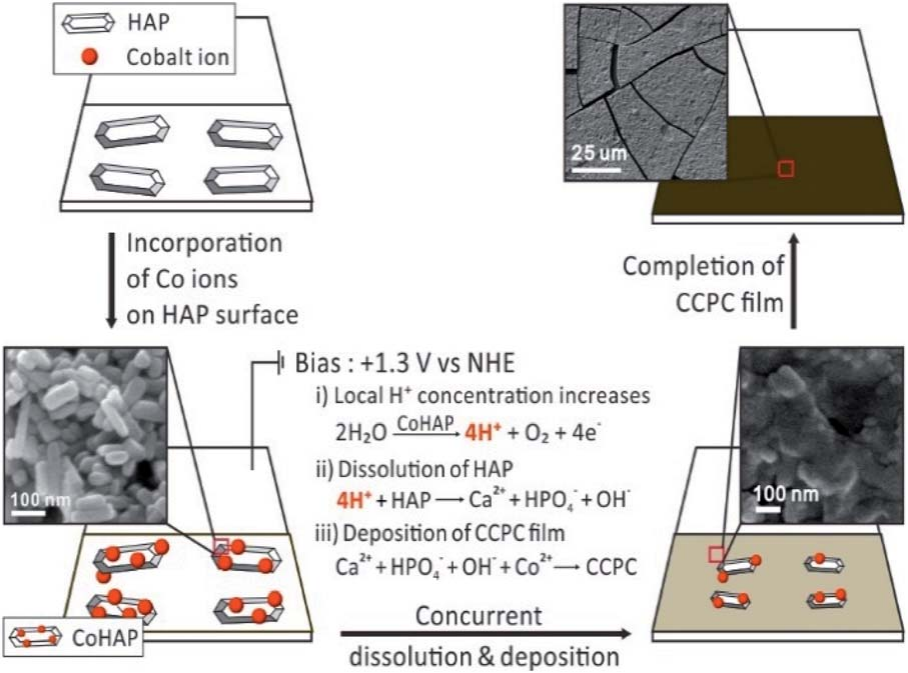
The process of CCPC film formation, the top three images represent early, middle, and last steps of SEM images during film formation.

Previously, we studied the surface characteristics and microscopic dissolution of HAP using *in situ* AFM.^19,20^ In addition, we prepared specific ions incorporated HAPs by immersing HAP into aqueous solution containing their ions, such as Co^2+^, Ag^+^ and F^−^.^21,22^ We found that the ion–exchange reaction or adsorption of metals (or metal oxide) takes place on the surface not into the bulk of HAP. Therefore, simple immersion of HAP into Co^2+^ solution (0.1 M) and Ag^+^ solution (0.1 M) (Fig. S7b and c†) does not induce the change of XRD patterns or the morphology of HAP. Bias driven CCPC formation is proposed in Scheme 1. First, immersing HAP on ITO into Co^2+^ solution induces the incorporation of cobalt ions on the surface of HAP (CoHAP). Unless biases (+1.3 V *vs.* NHE) is applied, hexagonal shape of HAP is preserved because incorporation of cobalt ions takes place on the surface of HAP, which is consistent to no change of HAP morphology by simple immersion into Co^2+^ solution (Fig. S4a and b†). Second, when the biases applied, the dissolution of HAP on ITO is proceeded by local increase of H^+^ ions caused by catalytic water oxidation of CoHAP. OER catalytic property of CoHAP is recently reported in neutral pH condition.^23^ Concurrently, calcium and phosphate ions produced from HAP dissolution recombine with cobalt ions or reactive oxygen species from water oxidation. Recombination of these ions results in precipitation of the CCPC film on the surface of ITO. Importantly, the recombination of these ions near the electrode surface are faster than the rate of ions diffusion into bulk solution. Consequently, the unwanted precipitation such as cobalt phosphate/oxide is prevented in the bulk solution. Finally, the CCPC film formation is terminated by the entire dissolution of HAP.

**Fig. 4:**
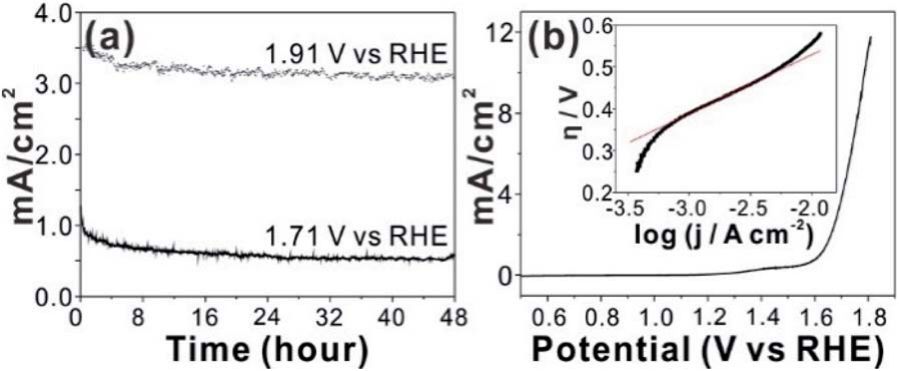
(a) OER durability of CCPC film on ITO (2 × 0.5 cm^2^) in 0.1 M KPi (pH 7) buffer at 1.91 V and 1.71 V *vs.* RHE, and (b) current–voltage characteristic curves of CCPC film on GC-RDE (diameter; 5 mm) in 0.1 M KPi buffer (pH 7) at 1600 rpm (inset; corresponding Tafel plot).

An *in situ* formed Co–Pi^4^ reported by Nocera group by anodic electrolysis in phosphate buffer has been widely investigated for OER catalyst. It was proposed that relatively small amount of phosphate group in Co-Pi plays key role OER catalytic property. In addition, various amorphous metal oxide/phosphates showed enhanced catalytic activity over crystalline counterparts.^24–26^ High density of local surface defects or coordinatively unsaturated metal centers presumably serve as catalytic active sites in amorphous materials. Because our CCPC film is basically composed of amorphous cobalt oxide with phosphate and calcium, we test OER catalytic properties. The CCPC film prepared on ITO evolves oxygen gas with high durability under pH 7 for 48 h (Fig. 4a and Movie S2†). In addition to ITO, the CCPC film can be formed on the glassy carbon electrode. Current–voltage characteristic shows the presence of oxidative wave at 1.0 V *vs.* NHE (inset in Fig. S8a†). This oxidation peak was previously reported in Co–Pi catalyst and originated from the oxidation state change of Co^2+^ to Co^3+^ (or Co^4+^).^27,28^ 556 mV of overpotential is needed to drive 10 mA cm^−2^ of the current density at 1600 rpm at pH 7 (Fig. 4b). This result is comparable to catalytic properties of other cobalt based catalysts near neutral pH condition.^4,29–34^Fig. 4b (inset) shows the Tafel plot of the CCPC film. Tafel plot is fitted to equation: *η* = *a* + *b* log *j* (*η* is overpotential, *b* is the Tafel slope, and *j* is current density). Tafel slope is almost same (144–150 mV dec^−1^) without dependence on rotating speeds (Fig. S8b†). Moreover, we introduce the CCPC film on Ni-foam (1 × 1 cm^2^) and evaluate its OER catalytic property in 1.0 M KOH solution (Fig. S9†). While bare Ni-foam needs 466 mV of overpotential to get 30 mA cm^−2^, the CCPC coated Ni-foam requires only 378 mV (Fig. S9a†). In addition, Tafel slope (Fig. S9b†) is improved after deposition of the CCPC film (144.2 and 126.5 mV dec^−1^ for bare Ni-foam and the CCPC coated Ni-foam, respectively). Multi-step chronopotentiometric curves of bare Ni-foam and the CCPC film are compared in Fig. S9c.† The potential level off at 1.56 V for the CCPC film and 1.63 V *vs.* RHE for Ni-foam at 5 mA cm^−2^. The CCPC film on Ni-foam needs lower potential than bare Ni-foam in entire current range (5–50 mA cm^−2^).

## Conclusions

In summary, we develop the novel method of electrochemical composite film having phosphate group using hydroxyapatite as phosphate source. To the best of authors' knowledge, solid phase of HAP has not been applied as phosphate source in electrochemical film preparation. The concurrent electrochemical dissolution of HAP and the deposition of the CCPC film occurs on the surface of electrodes without diffusion of phosphate ions into bulk solution. Consequently, unnecessary precipitation of metal phosphate is prevented. We demonstrate that the CCPC film exhibits OER catalytic properties in neutral pH as well as basic condition. Compared to OER catalytic properties of previous CoHAP studied by our group,^21^ CCPC film exhibit approximately 10 times improved current density. Additionally, our preparation method of the CCPC film is applicable to various substrates. Finally, we will further investigate the feasibility of film formation for other transition metal phosphates using HAP loaded electrodes.

## Conflicts of interest

There are no conflicts to declare.

## Supplementary Material

RA-011-D1RA05108C-s001

RA-011-D1RA05108C-s002

RA-011-D1RA05108C-s003

## References

[cit1] Cao G., Hong H. G., Mallouk T. E. (1992). Acc. Chem. Res..

[cit2] Nriagu J. O. (1984). Phosphate Minerals.

[cit3] Awad S. A., Kamel K. M. (1970). J. Electroanal. Chem..

[cit4] Kanan M. W., Nocera D. G. (2008). Science.

[cit5] Markevich E., Sharabi R., Gottlieb H., Borgel V., Fridman K., Salitra G., Aurbach D., Semrau G., Schmidt M. A., Schall N., Bruenig C. (2012). Electrochem. Commun..

[cit6] Li Y., Zhang L., Torres-Pardo A., González-Calbet J. M., Ma Y., Oleynikov P., Terasaki O., Asahina S., Shima M., Cha D., Zhao L., Takanabe K., Kubota J., Domen K. (2013). Nat. Commun..

[cit7] Barroso M., Cowan A. J., Pendlebury S. R., Grätzel M., Klug D. R., Durrant J. R. (2011). J. Am. Chem. Soc..

[cit8] Jegannathan S., Sankara Narayanan T. S. N., Ravichandran K., Rajeswari S. (2006). Surf. Coat. Technol..

[cit9] Bach S., Celinski V. R., Dietzsch M., Panthöfer M., Bienert R., Emmerling F., Schmedt Auf Der Günne J., Tremel W. (2015). J. Am. Chem. Soc..

[cit10] Sinha P. K., Feser R. (2002). Surf. Coat. Technol..

[cit11] Wang C. M., Liau H. C., Tsai W. T. (2006). Surf. Coat. Technol..

[cit12] Totik Y. (2006). Surf. Coat. Technol..

[cit13] Shanmugam S., Ravichandran K., Sankara Narayanan T. S. N., Lee M. H. (2015). RSC Adv..

[cit14] Kay M. I., Young R. A., Posner A. S. (1964). Nature.

[cit15] Kumar R., Prakash K. H., Cheang P., Khor K. A. (2004). Langmuir.

[cit16] Taş A. C. (2001). J. Am. Ceram. Soc..

[cit17] Xie L., Zhang R., Cui L., Liu D., Hao S., Ma Y., Du G., Asiri A. M., Sun X. (2017). Angew. Chem., Int. Ed. Engl..

[cit18] Ryu J., Jung N., Jang J. H., Kim H.-J., Yoo S. J. (2015). ACS Catal..

[cit19] Kwon K. Y., Wang E., Chung A., Chang N., Lee S. W. (2009). J. Phys. Chem. C.

[cit20] Kwon K. Y., Wang E., Chang N., Lee S. W. (2009). Langmuir.

[cit21] Jaworski J. W., Cho S., Kim Y., Jung J. H., Jeon H. S., Min B. K., Kwon K. Y. (2013). J. Colloid Interface Sci..

[cit22] Kwon K. Y., Wang E., Nofal M., Lee S. W. (2011). Langmuir.

[cit23] Pyo E., Lee K., Jang M. J., Ko I. H., Kim C. S., Choi S. M., Lee S., Kwon K. Y. (2019). ChemCatChem.

[cit24] Smith R. D. L., Prévot M. S., Fagan R. D., Zhang Z., Sedach P. A., Siu M. K. J., Trudel S., Berlinguette C. P. (2013). Science.

[cit25] Tsuji E., Imanishi A., Fukui K. I., Nakato Y. (2011). Electrochim. Acta.

[cit26] Wu G., Zheng X., Cui P., Jiang H., Wang X., Qu Y., Chen W., Lin Y., Li H., Han X., Hu Y., Liu P., Zhang Q., Ge J., Yao Y., Sun R., Wu Y., Gu L., Hong X., Li Y. (2019). Nat. Commun..

[cit27] McAlpin J. G., Surendranath Y., Dincã M., Stich T. A., Stoian S. A., Casey W. H., Nocera D. G., Britt R. D. (2010). J. Am. Chem. Soc..

[cit28] Ryu J., Jung N., Jang J. H., Kim H. J., Yoo S. J. (2015). ACS Catal..

[cit29] Ahn H. S., Tilley T. D. (2013). Adv. Funct. Mater..

[cit30] Kim H., Park J., Park I., Jin K., Jerng S. E., Kim S. H., Nam K. T., Kang K. (2015). Nat. Commun..

[cit31] Grzelczak M., Zhang J., Pfrommer J., Hartmann J., Driess M., Antonietti M., Wang X. (2013). ACS Catal..

[cit32] Ramsundar R. M., Debgupta J., Pillai V. K., Joy P. A. (2015). Electrocatalysis.

[cit33] Wei J., Feng Y., Liu Y., Ding Y. (2015). J. Mater. Chem. A.

[cit34] Xu K., Cheng H., Liu L., Lv H., Wu X., Wu C., Xie Y. (2017). Nano Lett..

