# Supplementary material for: Concurrent occurrence of electrochemical dissolution/deposition of cobalt–calcium phosphate composite
Source: RSC Adv. 2021 Aug 23;11(45):28342–6. doi: 10.1039/d1ra05108c (PMC9037991; doi:10.1039/d1ra05108c)
Supplement: RA-011-D1RA05108C-s003 [file RA-011-D1RA05108C-s003.pdf]

Electronic Supplementary Material (ESI) for RSC Advances.
This journal is © The Royal Society of Chemistry 2021

Supporting information

Concurrent Occurrence of Electrochemical Dissolution/Deposition of Cobalt-Calcium Phosphate Composite

Eunji Pyo,[a] Keunyoung Lee,[a] Gi-Tae Park,[b] Se-Young Ha,[b] Seonhong Lee,*[b] Chung Soo Kim*[b] and Ki-Young Kwon*[a]

[a]Department of Chemistry, Gyeongsang National University and Research Institute for Green Energy Convergence Technology, Jinju, 52828, South Korea

[b]Analysis & Certification Center, Korea Institute of Ceramic Engineering & Technology, Jinju, 52851, South Korea

Experimental details:

Materials. Calcium nitrate tetrahydrate ($Ca(NO_3)_2·4H_2O$, Duksan), sodium phosphate dibasic (Na_2HPO_4, Samchun, 99.0 %), cobalt nitrate hexahydrate ($Co(NO_3)_2·6H_2O$, Daejung), potassium phosphate dibasic (K_2HPO_4, Sigma Aldrich, >98 %), potassium phosphate monobasic (KH_2PO_4, Sigma Aldrich) and Nafion 117 solution (Sigma Aldrich) were purchased and used without purification.

Synthesis of Hydroxyapatite (HAP). HAP was synthesized by a hydrothermal method under strong basic condition. 30 mmol Na_2HPO_4 of 75 mL solution was added slowly for 10 minutes into 50 mmol $CaNO_3·4H_2O$ of 45 mL solution during vigorously stirring, and followed by addition of 20 mL of 10 M NaOH solution. After stirring the solution mixtures for 30 minutes in room temperature, the white color solution was transferred into a Teflon-lined autoclave reactor and maintained in a heating oven at 200 ^oC for 24 h. After cooling the solution at ambient condition, white precipitate was washed three times using distilled water and centrifuged. Finally, HAP was obtained by freeze-drying overnight.

HAP loaded on ITO glass. ITO (2×2 cm^2) was washed by sonicating in DI water, acetone and EtOH for 30 sec in each solvent and dried under Ar gas. HAP (5 mg), EtOH (150 μL) and Nafion 117 (5.4 μL) were sonicated for 30 min. 10 μL of HAP ink was dropped on a 2×0.5 cm^2 of the cleaned ITO glass and dried at ambient condition.

HAP loaded on glassy carbon electrode. Glassy carbon rotating disk electrode (GC-RDE, diameter; 5 mm) was polished by 0.05 μm alumina powder and sonicated 3 times using DI water for 20 sec and dried under Ar gas. 2 μL of the same HAP ink was dropped on the polished GC-RDE and dried at ambient condition.

HAP loaded on Ni-foam. Ni-foam (2×1 cm^2) was cleaned in 0.5 M of H_2SO_4 solution with sonication for 5 min and washed 3 times in DI water. The cleaned Ni-foam was immerged in HAP synthesis solution as described above. Ni-foam containing solution mixture was transferred into a Teflon-lined autoclave reactor and maintained in a heating oven at 200 ^oC for 24 hr. After cooling the solution, HAP loaded on Ni-foam was rinsed using 100 mL of DI water.

Electrodeposition of Cobalt-Calcium Phosphate Composite (CCPC) Film. The working electrodes were HAP loaded on substrates (ITO glass, GC-RDE and Ni-foam). The Ag/AgCl and Pt-plate were used as the reference and counter electrode, respectively. Electrochemical cell held 50 mL of 20 mM of aqueous cobalt nitrate solution. Electrolysis was carried out at +1.103 V vs Ag/AgCl for 3 hours without stirring.

Electrochemical Method. All electrochemical experiments were carried out a three-electrode electrochemical cell system. The CCPC coated substrates were used as the working electrodes. Ag/AgCl (BASi, 3 M KCl) for pH 7, Hg/HgO (1.0 M KOH) for pH 14 and Pt-plate (area: 5 cm^2, Wanatech) were applied as a reference and counter electrodes, respectively. Electrochemical experiments were conducted under ambient condition using commercial potentiostat (ZIVE SP1, Wonatech). The electrolytes were 0.1 M potassium phosphate buffer pH 7.0 (0.1 M KPi pH 7.0) for the CCPC coated on ITO or GC-RDE and 1.0 M KOH (pH 14) for the CCPC coated on Ni-foam. Potentials were converted to the normal hydrogen electrode (NHE) or reversible hydrogen electrode (RHE) using the following formula: E(NHE) = E(Ag/AgCl) + 0.197 (V), E(RHE) = E(NHE) + 0.059 (V) × pH.

Characterization. The crystalline phases of HAP and the CCPC film on ITO were determined by the grazing incidence X-ray diffraction (GIXRD, Smartlab, Rigaku). The prepared CCPC film morphology was characterized by a TITAN G2 60-300 transmission electron microscopy (TEM) equipped with CETCOR corrector and 4-silicon drift detector system energy dispersive X-ray spectroscopy operating at an accelerating voltage of 80 kV. Field emission scanning electron microscopy (FE-SEM) were obtained with a S8000 (TESCAN). The CCPC film underwent elemental analysis using an inductively coupled plasma atomic emission spectrometer (ICP-AES, OPIMA 4300DV/5300DV, Perkin Elmer) to identify the elemental composition of cobalt, calcium and phosphorous. The XPS results were obtained on a PHI 5000 Versaprobe instrument (ULVAC-PHI Inc., Chigasaki, Japan) in which monochromated Al Kα radiation (hv=1486.6 eV) was illuminated as the light source.

Current-Voltage characteristic. The CCPC coated on GC-RDE was scanned by applying the potential of 0 ~ 1.3 V vs Ag/AgCl at a scan rate of 5 mV/s in 0.1 M KPi (pH 7) at 900, 1600 and 2500 rpm with iR compensation (85 % of iR-drop was compensated by the potentiostat). The CCPC coated on Ni-foam was tested by applying potential of 0 ~ 0.8 V vs Hg/HgO at a scan rate of 5 mV/s in 1.0 M KOH (pH 14) without iR compensation.

Durability Test of the CCPC film on ITO. The potentials were tested under two conditions, 1.103 V and 1.303 V vs Ag/AgCl, in 0.1 M KPi buffer (pH 7) and the potentials were applied for 48 hr without iR compensation.

Multi-step chronopotentiometric. The multi-step chronopotentiometric of CCPC coated Ni-foam was proceeded in 1.0 M KOH (pH 14). The current density started at 5 mA/cm^2 and ended at 50 mA/cm^2, with an increasement of 5 mA/cm^2 per 20 min without iR compensation.

Tafel plot. Tafel slope was calculated by the data of linear sweep voltammetry (LSV) in 0.1 M KPi (pH 7) buffer and 1.0 M KOH (pH 14). The overpotential was calculated using the following equation.

$$\eta = applied\ potential\ (V)\ vs\ NHE - (1.23\ V - 0.059\ V\ \times (pH\ 7\ or\ 14))$$

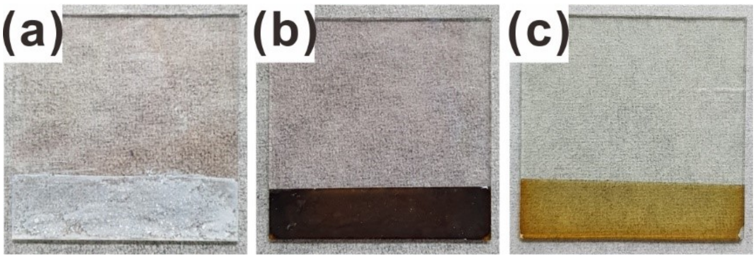

Figure S1. The photographs of the (a) HAP, (b) CCPC film, (c) electrodeposited Co^{2+} on bare ITO glass.

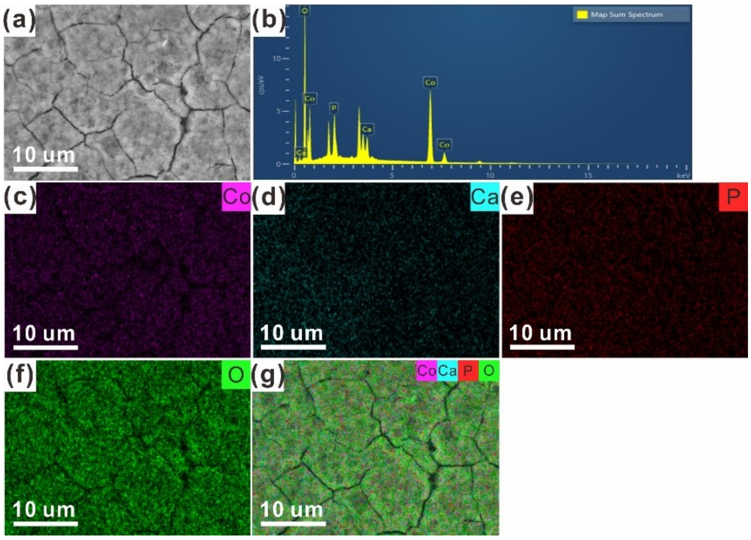

		At.%			
		Co	Ca	P	O
Sample 1	1	23.38	3.48	4.52	68.62
	2	24.28	3.23	4.65	67.84
	3	24.67	3.43	4.86	67.04
	4	27.86	3.54	5.13	63.48
	5	23.44	3.52	4.46	68.57
	Average	24.726	3.44	4.724	67.11
Sample 2	1	24.65	3.08	4.83	67.44
	2	24.12	3.05	4.59	68.24
	3	25.92	2.94	4.85	66.29
	4	26.85	3.02	4.9	65.23
	5	24.11	2.88	4.6	68.41
	Average	25.13	2.994	4.754	67.122

Figure S2. SEM-EDX elements mapping of the CCPC film (a) SEM image, (b) EDX spectroscopy, (c) Co, (d) Ca, (e) P, (f) O and (g) Overlapping of Co, Ca, P and O. The atomic % of Co, Ca, P and O of two CCPC films are listed on the table (right).

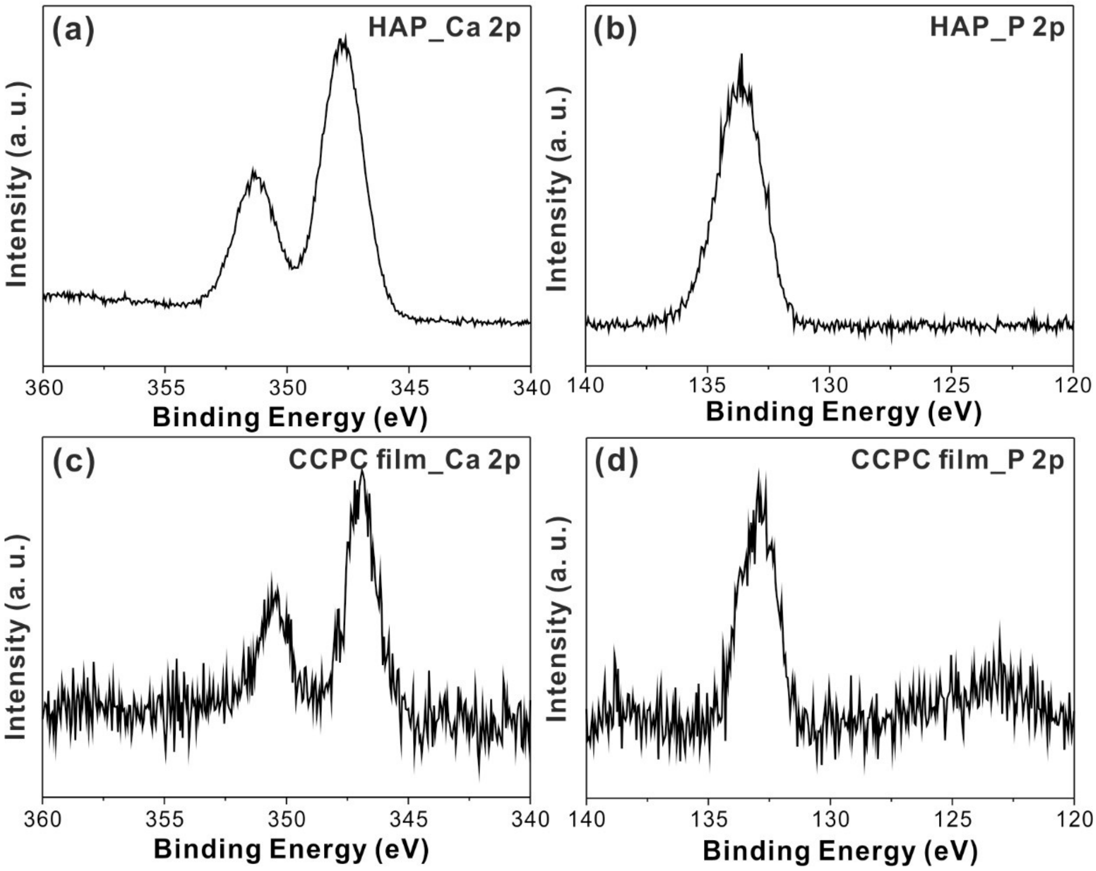

Figure S3. XPS spectra of (a and b) HAP and (c and d) CCPC film, (a and c) Ca 2p and (b and d) P 2p.

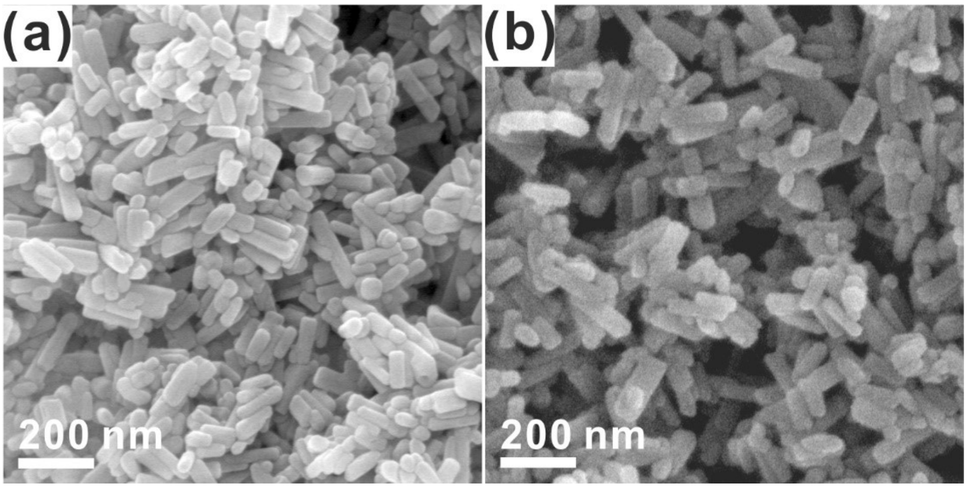

Figure S4. SEM images of (a) HAP and (b) HAP immersion into aqueous Co^{2+} solution (20 mM) after 3 hr.

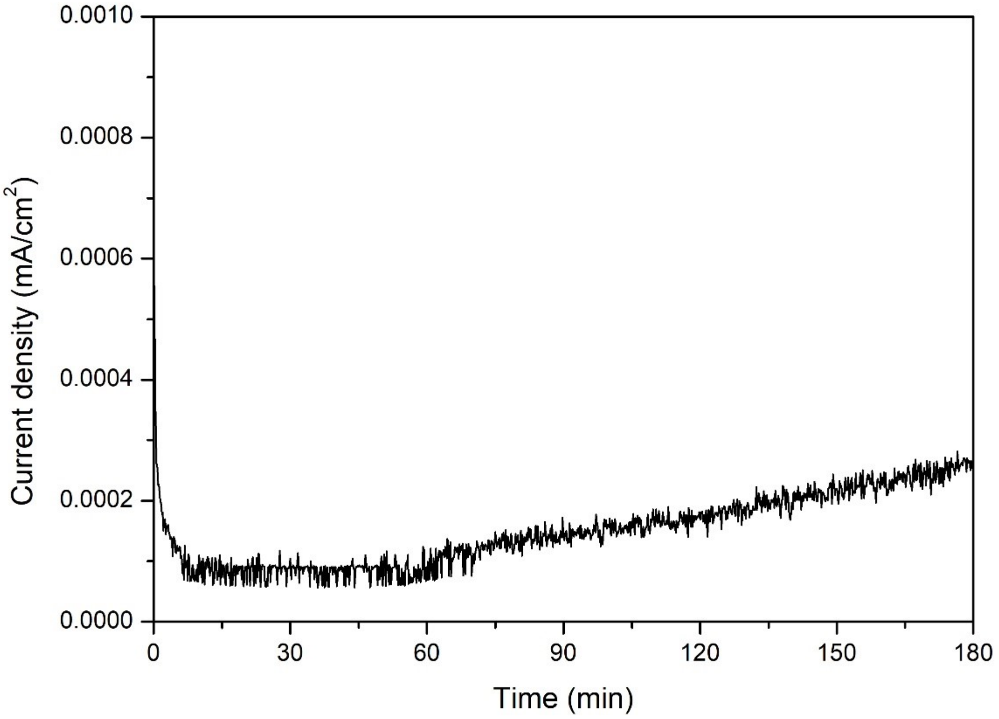

Figure S5. Current profile of HAP loaded on ITO glass in 20 mM $NaNO_3$ solution at +1.3 V vs NHE for 3 hours.

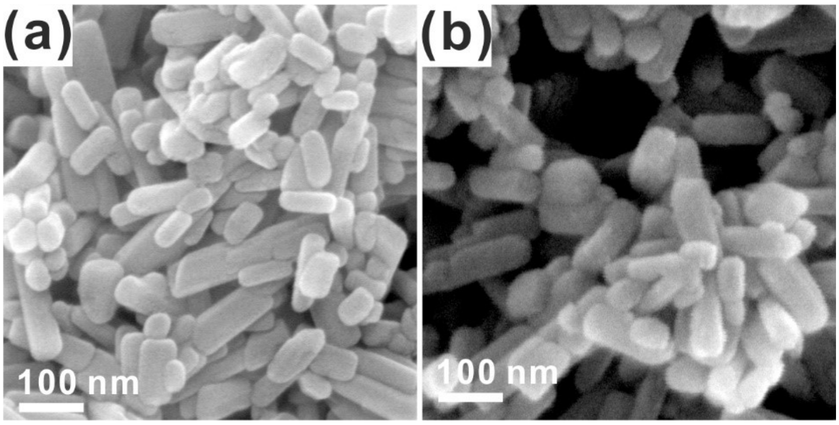

Figure S6. SEM images of (a) HAP and (b) HAP after anodic electrolysis at +1.3 V vs NHE in 20 mM $NaNO_3$ solution.

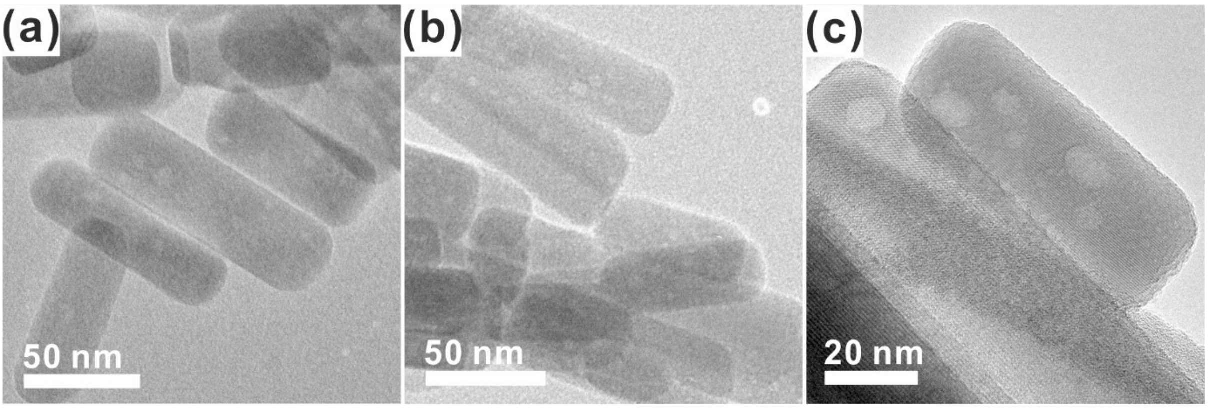

Figure S7. TEM images of (a) HAP, (b) Ag incorporated HAP and (c) Co incorporated HAP. By simple immersing of HAP into Co^{2+} solution (0.1 M) and Ag^{+} solution (0.1 M), no morphological change is observed (Fig. S7b and c).

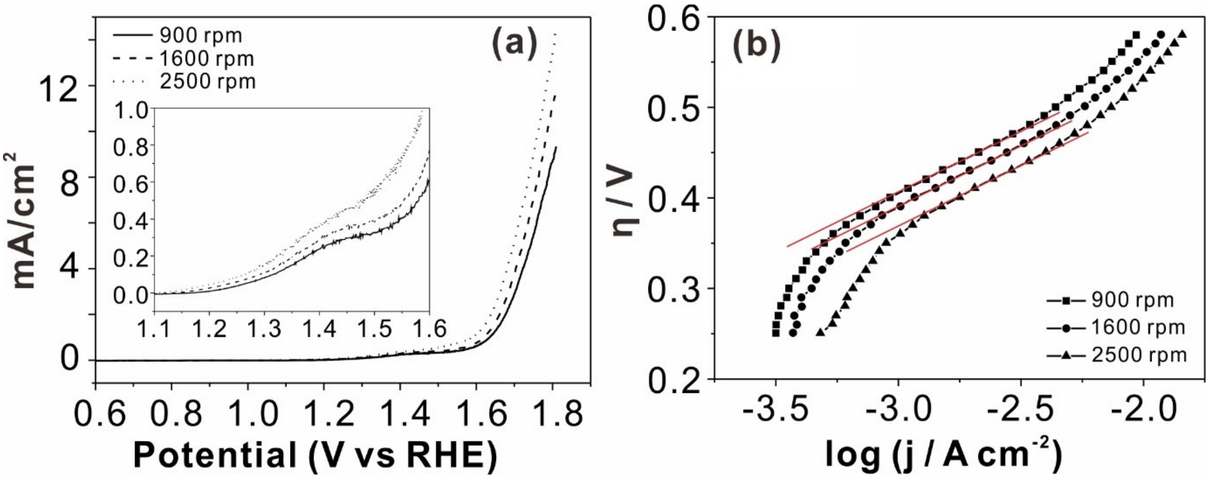

Figure S8. (a) Current-Voltage characteristic curves of CCPC film on GC-RDE (insert; cobalt oxidative wave) and (b) Tafel plots of CCPC film on GC-RDE at different rotating speeds.

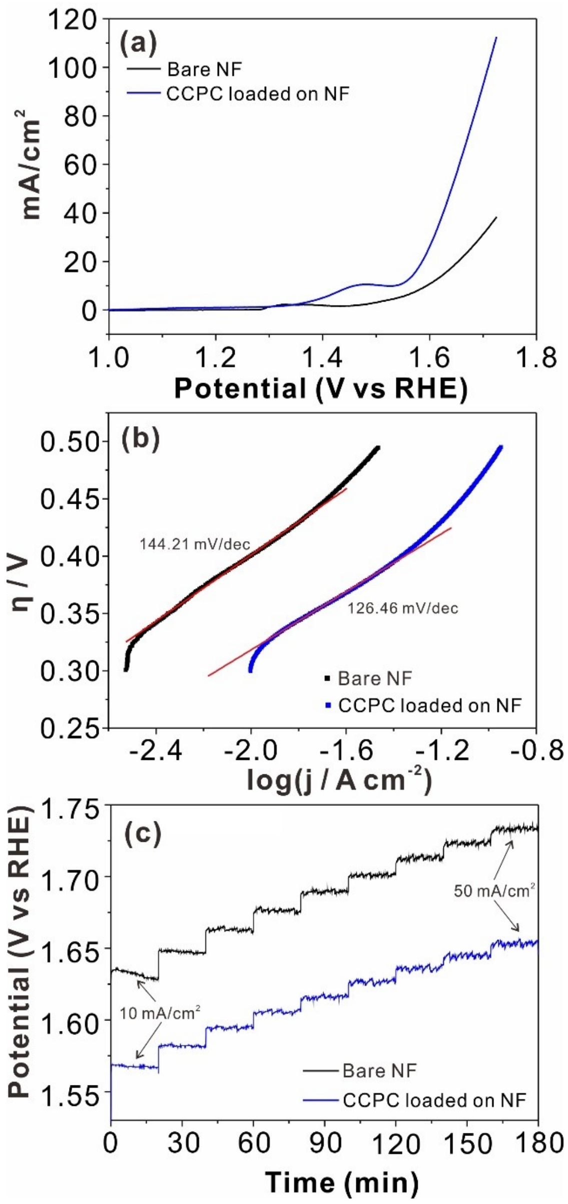

Figure S9. (a) Current-Voltage characteristic curves for bare Ni-foam and CCPC loaded on Ni-foam with a scan rate of 5 mV/s in 1.0 M KOH (pH 14) without iR compensation, (b) Corresponding Tafel plots for bare Ni-foam and CCPC loaded on Ni-foam, (c) Multi-current process of bare Ni-foam and CCPC loaded on Ni-foam in 1.0 M KOH. The current density started at 5 mA/cm^2 and ended at 50 mA/cm^2, with an increment of 5 mA/cm^2 per 20 min.

Movie S1. The entire process of in-situ formation of the CCPC film in 20 mM aqueous Co^{2+} solution at +1.3 V vs NHE for 90 min.

Movie S2. Bulk electrolysis of CCPC film on ITO at +1.91 V vs RHE in 0.1 M KPi buffer (pH 7) solution.
